# 6.C. The revised EU health security framework

**DOI:** 10.1093/eurpub/ckac129.352

**Published:** 2022-10-25

**Authors:** Organised by: DG SANTE and ECDC, Chair persons: Isabel de la Mata (European Commission - DG Sante)

## Abstract

As a response to the pandemic, the Commission's communication of 11 November 2020 proposed building blocks for a European Health Union (EHU) and advocated the strengthening of existing structures and mechanisms for better EU level protection, prevention, preparedness and response against human health hazards. It recommended a reinforced framework for cross-border cooperation against all health threats to protect better human lives and the internal market as well as to maintain the highest standards in the protection of human rights and civil liberties, and a strengthened EU role in international cooperation to prevent and control cross-border health threats and improve global health security. By upgrading the EU health security framework for cross-border health threats, the building blocks of the European Health Union bring greater overall impact while fully respecting the Member States’ competence in the area of health. Concretely, before the end of 2022, two pieces of legislation will have been adopted: the upgrading of Decision 1082/2013/EU and the strengthened mandate of the European Centre for Disease Prevention and Control (ECDC) and the European Medicines Agency (EMA) that go hand in hand. The new Regulation on serious cross-border threats to health, the overarching legislative piece of the EU Health Union Package, aims at a stronger and more comprehensive legal framework for the Union to better prepare and respond to serious cross-border threats to health and public health emergencies. It better governs actions at Member States and Union level on prevention, preparedness and response, surveillance, risk assessment, and early warning. Further, it ensures better arrangements for joint procurement at EU level. With its revised mandate, the ECDC will be able to issue recommendations to Member States regarding preparedness and response, host a new network of EU reference laboratories and establish an EU Health Task Force for crisis preparedness and rapid public health interventions in case of a major outbreak. The regulation reinforcing EMA's role in crisis preparedness and management of medicinal products and medical devices puts some of the structures and processes established during the COVID-19 pandemic on a permanent footing. EMA is now responsible for monitoring medicine shortages, as well as reporting shortages of critical medicines during a crisis. It will also coordinate responses of EU / EEA countries to shortages of critical medical devices and in-vitro diagnostics in crises. The EU Health Security Framework will link up to IHR (where EU is requesting to become a contracting party) and the pandemic treaty, and the parallel developments to improve pandemic preparedness and response in G7 and G20 framework (also new global health strategy). The revised legislation addresses the weaknesses evidenced by the COVID-19 pandemic and support actions that can be financed via the EU4Health programme and other EU funding instruments. DG SANTE and ECDC will present the new legislation; two Member States will share their views on the newly adopted legislation. HaDEA will present the funding opportunities through EU4Health to support the implementation of th

e two new legislation.

**Speakers/Panellists:**

**Ingrid Keller**

European Commission, Luxembourg, Luxembourg

**Florina Telea**

European Commission, Brussels, Belgium

